# De Novo Nucleic Acids: A Review of Synthetic Alternatives to DNA and RNA That Could Act as Bio-Information Storage Molecules †

**DOI:** 10.3390/life10120346

**Published:** 2020-12-11

**Authors:** Kevin G Devine, Sohan Jheeta

**Affiliations:** 1 School of Human Sciences, London Metropolitan University, 166-220 Holloway Rd, London N7 8BD, UK; K.Devine@londonmet.ac.uk; 2 Network of Researchers on the Chemical Evolution of Life (NoR CEL), Leeds LS7 3RB, UK

**Keywords:** non-standard nucleic acids, sugar-phosphate backbone, pentose sugars, hexose derivatives, phosphate group replacement, alien life forms

## Abstract

Modern terran life uses several essential biopolymers like nucleic acids, proteins and polysaccharides. The nucleic acids, DNA and RNA are arguably life’s most important, acting as the stores and translators of genetic information contained in their base sequences, which ultimately manifest themselves in the amino acid sequences of proteins. But just what is it about their structures; an aromatic heterocyclic base appended to a (five-atom ring) sugar-phosphate backbone that enables them to carry out these functions with such high fidelity? In the past three decades, leading chemists have created in their laboratories synthetic analogues of nucleic acids which differ from their natural counterparts in three key areas as follows: (a) replacement of the phosphate moiety with an uncharged analogue, (b) replacement of the pentose sugars ribose and deoxyribose with alternative acyclic, pentose and hexose derivatives and, finally, (c) replacement of the two heterocyclic base pairs adenine/thymine and guanine/cytosine with non-standard analogues that obey the Watson–Crick pairing rules. This manuscript will examine in detail the physical and chemical properties of these synthetic nucleic acid analogues, in particular on their abilities to serve as conveyors of genetic information. If life exists elsewhere in the universe, will it also use DNA and RNA?

## 1. Introduction

Life on Earth uses three key biopolymers, namely nucleic acids, proteins and polysaccharides; each of which possesses intrinsic structural features. Nucleic acids are polymers comprised of heterocyclic aromatic bases appended to a sugar-phosphate backbone, held together by phosphodiester bonds. Proteins are polymers of amino acids linked via amide bonds of dubbed as peptide bonds [−C(=O)N(−H)−] and polysaccharides are polymers of carbohydrates linked via acetal ether bonds. These unique chemical features have long fascinated organic chemists and stimulated the most creative minds among them to question nature’s choices, and, indeed, design and test alternatives using the power of laboratory-based synthetic organic chemistry. This paper will focus entirely upon re-designed nucleic acids, which feature three key structural modifications of their natural counterparts: (a) replacement of the phosphate moiety with an uncharged analogue, (b) replacement of the pentose sugars ribose and deoxyribose with alternative acyclic, pentose and hexose derivatives and, finally, (c) replacement of the two heterocyclic base pairs adenine/thymine and guanine/cytosine with non-standard analogues that obey (or disobey) the Watson–Crick pairing rules. As will be shown, the results are indeed intriguing and have profound consequences for the development of artificial Darwinian chemical systems, and the discovery of life, if it exists, elsewhere in the Universe. This is an example of how the *synthesis paradigm* can drive discovery and understanding in ways that *analysis* of the natural world alone cannot. Just as the most skilled mechanical, electrical and software engineers can build modern automobiles, aircraft and super-computers, creations whose intricate inner workings they fully understand, so, it is hoped, the new generation of synthetic biologists will be able to manufacture, and thus fully understand, artificial life forms, built from different biopolymers to those found in nature.

### 1.1. Nucleic Acid Structure

**Figure 1:**
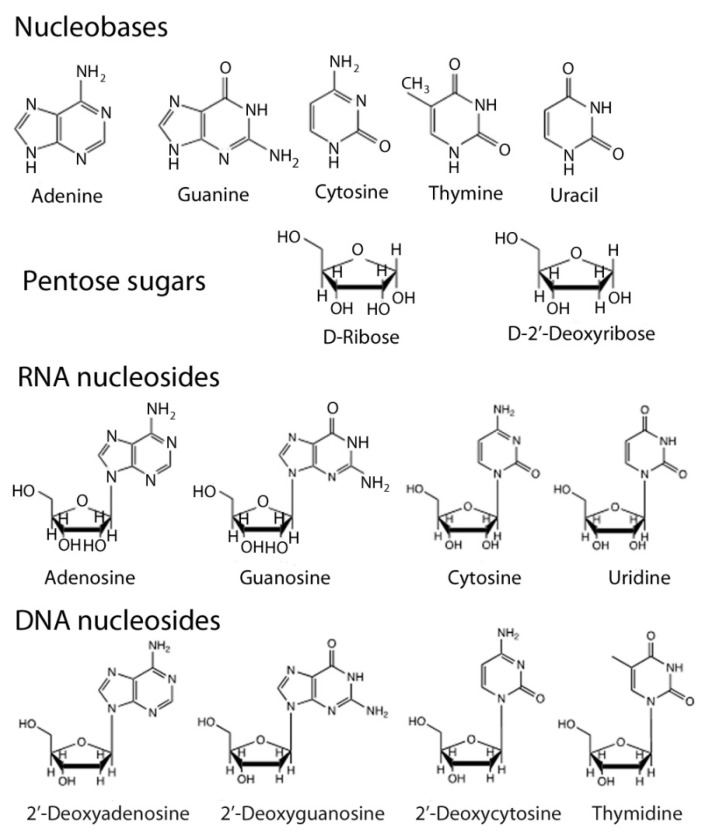
The molecular structures of the nucleobases, sugars and nucleosides found in DNA and RNA.

Nucleic acids are biopolymers that are built from nucleotides. The latter consist of three molecular components; a heterocyclic aromatic base (also known as a nucleobase), a five-atom ring sugar, and a phosphate unit that connects the sugars together and forms the alternating sugar-phosphate backbone. The structures of the nucleobases, the two sugars and the nucleobase-sugar conjugates, which are known as nucleosides, are shown in Figure 1. DNA differs from RNA in two distinct ways: the sugar is 2′-deoxyribose instead of ribose, and the pyrimidine base thymidine has a methyl group attached at the 5-position where uracil has hydrogen and so technically speaking the thymidine is a 5′-methyluracil base.

The numbering systems are distinct for the nucleobases and sugars, with the latter using affixed “primed” numbers 1′-5′ to distinguish them (Figure S1a, in the Supplementary Materials). Oligonucleotides, or polynucleotides, are polymers made from nucleosides that are linked via their 3′- and 5′-oxygen atoms by phosphate groups (Figure S1b, in the Supplementary Materials). The sequence of bases is read from the 5′-end to the 3’-end (i.e., 5′ → 3′).

**Figure 2:**
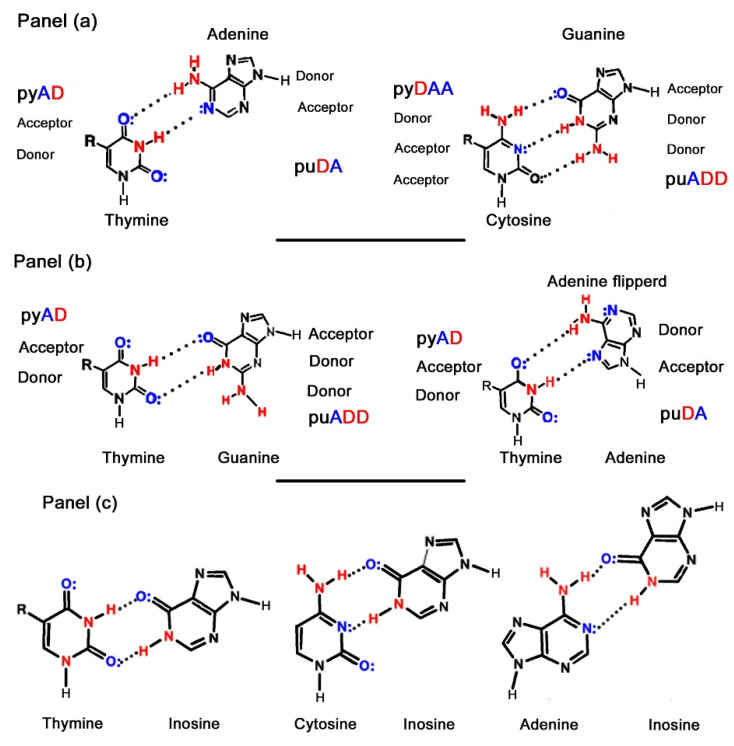
Panel (**a**): the base-pairs between T–A and C–G revealing both the size and hydrogen-bonding complementarities displayed by these pairs. Panel (**b**): The wobble base-pairing of T and G (left), and the Hoogsteen base-pairing of T and A (right). Panel (**c**): wobble base-pairings between thymine and inosine (left), cytosine and inosine (centre), and adenine and inosine (right).

The base sequence of an oligonucleotide is important because genetic information is stored in the sequence of these bases in a DNA (or RNA) molecule. The key storage unit for genetic information in most organisms is not a single-stranded DNA oligomer, instead, it is two complementary strands. These strands are held together by base-pairs on opposite strands which follow two complementary principles: size and hydrogen-bonding complementarity. In size complementarity, a large 9-atom bicyclic purine base (adenine and guanine) pairs with a small, 6-atom ring pyrimidine base (uracil/thymine and cytosine). In hydrogen-bonding complementarity, hydrogen bond donors (N-H bonds) on one base interact with the hydrogen bond acceptors generally with lone pairs of electrons on N or O atoms on its partner in the opposite strand. In this way, an adenine on one strand pairs with a thymine (or uracil in RNA) in another, and a guanine likewise pairs with a cytosine (Figure 2 panel a).

**Figure 3:**
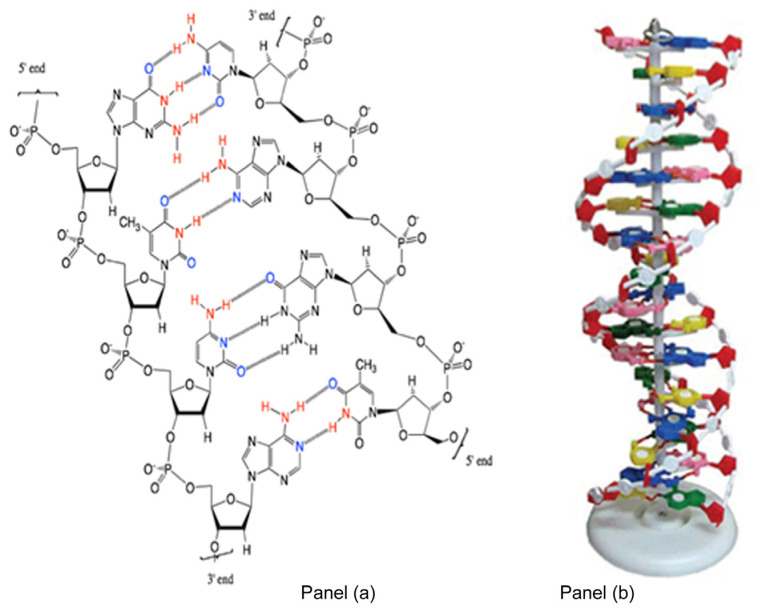
The duplex between two Watson–Crick base-paired complementary DNA strands panel (**a**), is arranged as a double-helix in which the base-pairs are the steps and the sugar-phosphate backbone the handles panel (**b**).

Two complementary strands held together by their base-pairs is known as a duplex. The two complementary single strands are oriented “anti-parallel” with respect to each other; a strand whose direction is 5′→3′ pairs with its’ 3′→5′ complement. The duplex nature of DNA was first revealed by the X-ray crystallographic data obtained by Rosalind Franklin and Maurice Wilkins in 1953. From this data, Francis Crick and James Watson were able to construct models, which showed that the two complementary strands were held together by hydrogen bonds between base partners, forming a double-helical spiral staircase in which the base-pairs are the steps and the sugar-phosphate backbone the handles [1]; Figure 3 panels a and b). The T–A and C–G hydrogen bonding interactions thus became known as Watson–Crick base-pairs, and their elucidation of the double helix nature of DNA earned them the 1964 Chemistry Nobel Prize, along with Maurice Wilkins (Rosalind Franklin had by then passed away in 1958).

Two non-Watson–Crick base pairings also occur and are known respectively as wobble and Hoogsteen pairings (Figure 2 panel b). The “wobble” pairs occur between thymine/uracil and guanine, thymine and inosine, cytosine and inosine and adenine and inosine. Inosine, which occurs only in transfer RNA (tRNA), is an analogue of guanosine that lacks the 2-NH_2_ and its wobble pairings help maintain the 3-D structure of tRNAs and explain the non-specificity of the genetic code (Figure 2 panel c).

Hoogsteen base-pairing occurs between thymine and adenine in which the N–H of thymine interacts with the N-7 of adenine, with the latter having “flipped” through 180° [2]. The biological significance of Hoogsteen pairings has still not yet been fully elucidated, but they may be important for stimulating mutations and for the stabilization of tRNA conformations [3].

It takes much more than just hydrogen-bonded base-pairing to hold the double-helical DNA duplex together. The base pairs are aromatic heterocycles and as such, are relatively hydrophobic. They interact with each other through a complex combination of van der Waals and electrostatic intermolecular forces known as “base-stacking” and thus readily “stack” on top of each other (Figure 3 panel b). This “base-stacking” removes these hydrophobic components away from water and into a more hydrophobic environment; this process is entropy-driven and drives duplex formation.

There are two major regular DNA duplex structures known as A-DNA and B-DNA. B-DNA is the form that predominates in the cellular nucleus and has ten base pairs per turn of the helix with the base pairs perpendicular to the helix axis. Two distinct structural features can be seen, a wide major groove and a narrow minor groove. A-DNA is a more tightly packed structure with 11 base pairs per helical turn and these base pairs are tilted at a 20° angle to the helix axis. In A-DNA, the major groove is narrow but deep, and the minor groove broad and shallow. Another type of DNA and RNA duplex conformer, known as Z-DNA and Z-RNA, was initially found in crystallised oligomers that contained alternate G–C sequences, in solutions containing high concentrations of salts (NaCl, MgCl_2_) or ethanol. Z-DNA is a left-handed double helical structure with ten base pairs per turn, in which the base pairs are roughly parallel to the helix axis. The phosphate backbone has a zig-zag appearance, and the minor groove is very deep and narrow, and the major groove extremely shallow. Recent studies have revealed the role of Z-DNA and Z-RNA in human diseases [4].

Moreover, both DNA and RNA possess a polyanionic backbone in which a negative charge is located on a phosphate oxygen, and there are seven atoms between each charge. This repeating polyanionic array renders all nucleic acids highly water-soluble, and gives them their predominant physical property, as Coulombic repulsions prevent intra-strand interactions and folding. The nucleic acids tend to stretch out in solution to minimize intra-strand repulsions, and this intrinsic property enables duplex formations to occur between two complementary strands; the relatively stretched single strands, upon interaction, can then arrange themselves into the minimum energy duplex conformation, driven by base-stacking, in which Watson–Crick base-pairings occur.

Furthermore, the polyanionic backbone dominates the physico-chemical properties of nucleic acids to such an extent that they are, essentially, independent of their base sequences. All nucleic acids containing the same number of bases (and hence negatively charged phosphates) will, regardless of their base sequences, move along a gel in an applied electric field at the same rate towards the anode. This behaviour is unique to nucleic acids and is what renders them fit to act as genetic polymers, which enable Darwinian evolution to take place.

### 1.2. Genetic Molecular Constraints

For any biopolymer to act as an efficient chemical storage system and conveyor of genetic information, it must have the following two important properties: first, it must be readily replicable, through the use of protein enzymes as in modern biology, and ribozymes or “self-replicating RNAs” in the precursor RNA world; second, it must be within a low tolerable rate of mutations; structural changes cannot compromise replication fidelity. In summary, a genetic biopolymer must be “Capable of Surviving Modifications in Constitution without Loss of Properties Essential for Replication” and this statement generates a very appropriate acronym: COSMIC-LOPER [5].

COSMIC-LOPER behaviour is unique to nucleic acids. In DNA and RNA, changing the base sequence does not change either the physical properties or chemical reactivity. This is certainly not the case for proteins, where changing even one amino acid in a protein sequence profoundly alters both the physical properties and the reactivity. Perhaps the best-known example of this is haemoglobin, a four-chain quaternary protein with four haem units responsible for the transport of molecular oxygen in the blood of vertebrates. A mutation which replaces the hydrophilic amino acid, glutamate, with the hydrophobic, valine, at position 6 in the B1 chain results in a much less soluble protein and is responsible for the debilitating disease of sickle cell anaemia.

In the enzyme ribonuclease, which cleaves RNA molecules, substitution of any of the four catalytic amino acids histidines at positions 12 and 119; lysine at position 41, and aspartic acid at position 121 renders this enzyme completely inactive.

### 1.3. Catalytic Nucleic Acids and the Origin of Life

An examination of contemporary biology encounters a classical “chicken and egg” paradox. Polymeric nucleic acids may contain all the information required to construct a protein, but proteins called polymerases are needed to catalyse nucleic acid synthesis. The big question is, how did it all begin? Did proteins and nucleic acids arise and encounter each other spontaneously (which is highly unlikely based upon statistical probability), or was there an initial biopolymer that could perform both catalysis and replication, in other words, was the first living entity a biopolymer that was self-replicating? Life can be defined as “a self-sustaining chemical system capable of undergoing Darwinian evolution”. This clearly distinguishes living chemistry from other physico-chemical processes like crystal growth. In the latter, for example, the addition of small crystals of sodium chloride to a saturated solution of this salt can seed the formation of more crystals. This may be considered as reproduction, but this system cannot undergo Darwinian evolution as there is no possible way that the structure of sodium chloride can be changed (by replacing either the sodium or the chloride ions) without interfering with the seeding of more crystals of this salt from solution. What is essential to the process of Darwinian evolution is that a small change needs to occur in structures, which may enable the replicating process to improve by just a tiny amount in future progeny. Conversely, such changes may result in the demise of the same structures. These small changes lie at the heart of the concept of Darwinian evolution, without which life on Earth is not possible, as exemplified by the non-biological replicator, sodium chloride above. These changes are often referred to as selection pressures and are due to ever-fluctuating environmental challenges like changes in temperature, pressure and/or pH.

Supporters of the “one-biopolymer” as the earliest form of life were given a big boost in the early 1980s with the discovery that RNA, in addition to acting as a chemical code repository (*cf* mRNA and genomes of Retroviruses), exhibits catalytic behaviour; for this discovery, Sydney Altmann [6,7,8] and Tom Cech [9] received the 1990 Nobel Prize in Chemistry. The catalytic activity of RNA molecules, referred to as “ribozymes” (*cf* enzymes), included cleavage and splicing of RNA inter-nucleotide bonds, as well as, more importantly, peptide bond formation.

Elucidation of the structure of the ribosome has revealed that protein synthesis, via peptide bond formation, is entirely carried out by non-coding ribosomal RNA (rRNA) molecules (*cf* mRNA, which is referred to as coding). The proteins present in the ribosome appear to act as scaffolds. This has led to the idea that an “RNA world”, whose organisms were effectively self-replicating RNA molecules followed later by organisms whose genomes consisted of RNA that was synthesized and processed entirely by proto-ribozymes, preceded the contemporary DNA-protein system.

Proteins are of course much better catalysts than ribozymes on account of the richer and more diverse functionalities to be found on their amino acid side chains, and the absence of a charged backbone, which tends to impede close-knit folding as a result of Coulombic repulsion. Ribozymes also require relatively high divalent metal ion concentrations (Mg^2+^, Mn^2+^, Pb^2+^) to function effectively. In addition to bringing reacting groups close together through ligand-binding, it is also believed that the high ionic strength is a prerequisite to overcome the Coulombic repulsion between the phosphate anions in the backbone, and thus enable a degree of folding, similar to that observed in tRNAs though unseen in genomic DNA and RNA.

### 1.4. Catalytic DNA Molecules: DNAzymes (Deoxyribozymes)

**Figure 4:**
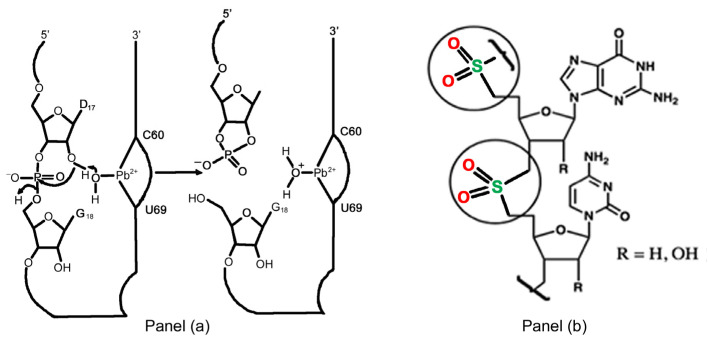
Panel (**a**): a proposed mechanism for the cleavage of a phosphodiester bond in yeast phenylalanine tRNA. The Pb^2+^ is bound to a specific site on the RNA around the bases U59 and C60. The active species is probably (PbOH)^+^ which has a pKa of about 7.0. Panel (**b**): oligosulfones, which contain a dimethylene sulfone group in place of phosphate, no longer form duplexes with Watson–Crick base-pairings but bend and fold more like proteins.

In 1994, the first catalytic DNA molecules, DNAzymes or deoxyribozymes, were reported by Breaker and Joyce [10]. These catalysts are purely artificial and are produced using a technique known as “Selective Evolution of Ligands by Exponential Enrichment (SELEX)”. Most deoxyribozymes are single-stranded 30–60 mer DNA molecules and have found widespread applications as catalysts for the cleavage and ligation of phosphodiester bonds in RNA, DNA and amino acid substrates (serine and tyrosine-phosphates); cleavage and ligation of ester bonds, and even a Diels–Alder cycloaddition reaction [11]. Like ribozymes, deoxyribozymes require relatively high divalent metal (e.g., Pb^2+^) salt solutions for optimal efficiency (Figure 4 panel a).

## 2. Synthetic Organic Modifications of Nucleic Acid Structure

During the past three decades, leading organic chemists have synthesized novel nucleic acids and studied their properties. These novel nucleic acids differ from their natural counterparts in three key features: replacement of the polyanionic backbone with uncharged analogues; replacement of the ribose and 2′-deoxyribose sugars with alternative acyclic, pentose and hexose derivatives; and replacement of the standard base-pairs adenine-thymine and guanine-cytosine with non-standard analogues, including some that don’t obey the two “complementarity” principles observed in DNA and RNA.

### 2.1. Nucleic Acids with Uncharged Backbones

The initial need for nucleic acid analogues with uncharged backbones was driven by a new therapeutic concept in the mid to late 1980s; the “anti-sense” approach, whose idea was straightforward and logical [12]. Many diseases, in particular cancer and viral infections such as AIDS and SARS, arise from the presence of unwanted DNA (oncogenes or viral DNA sequences) that are transcribed into unwanted messenger RNA, which gets translated into unwanted proteins. Most current drugs work by targeting and binding to undesired proteins. The anti-sense concept went back a stage further and targeted the undesired mRNA that preceded the undesired protein. Often the sequence of the undesired mRNA is known, so the best entity for binding would obviously be a single-stranded oligonucleotide complementary to a sequence of 15–20 bases on the mRNA, as the probability of such a sequence occurring anywhere else in the human genome is about one in ten trillion (and noting that there are only 3 billion base-pairs in the human genome). The resulting mRNA/oligonucleotide complex cannot interact with tRNA molecules on the ribosome, translation is therefore impeded, and RNase enzymes degrade the complex, so the undesired protein is not synthesised.

Administering a natural DNA molecule that is complementary to the 20 base sequence on the mRNA (referred to as an anti-sense molecule because it interacts with the coding sequence on mRNA which is a copy of the “sense” strand of the original gene sequence on DNA) has one severe drawback; human serum contains enzymes known as nucleases, which degrade DNA, so it is unlikely that enough of the DNA oligonucleotide would reach its target cell. It is the phosphodiester backbone, which is the target of these enzymes. Many researchers believed that the answer was to make analogues of DNA in which the sugar-phosphate backbone was replaced with neutral, often non-polar analogues, which would withstand degradation by nucleases. Much of this work began in the mid to late 1980s, before work performed by Steven Benner and others realized the importance of retaining a polyanionic backbone to preserve the Watson–Crick recognition properties of new oligonucleotide analogues. Many different backbones were made, and virtually all failed to show sequence-specific base pairing, the pre-requisite for use as antisense agents.

In the early to mid-1990s, work performed in Steven Benner’s laboratories, (both in Florida and previously at the ETH in Zurich, Switzerland) has revealed the importance of the polyanionic backbone. In a remarkable feat of skilled organic syntheses rivalling that of any complex natural product, Benner’s group constructed nucleic acid analogues in which the charged phosphate $\left[-O-P\left(=O\right)O^{-}-O-\right]\;$ is replaced by a polar but uncharged dimethylene sulfone $\left[-CH_{2}-SO_{2}-CH_{2}-\right]$ function [13]. These “oligosulfones” possess physico-chemical properties that are very different from their natural counterparts. In particular, they can bend and fold up in a manner more analogous to proteins, disrupting both base-stacking and Watson–Crick hydrogen bonding between two complementary strands, which, significantly, will not form duplexes (Figure 4 panel b). There are other examples in the literature where other groups have, likewise, replaced the charged phosphates with uncharged groups and observed similar results. This is a classic example of how a quintessential “blue skies” research enterprise could have guided and informed an important “bio-medicinal” one.

One notable exception to the observed properties of uncharged nucleic acid analogues like oligosulfones was displayed by the peptide nucleic acids (PNAs), developed by Peter Nielsen and his co-workers at the Panum Institute in Copenhagen, Denmark in the early 1990s [14]. These analogues differed significantly from their natural counterparts. The sugar-phosphate backbone was replaced by a linear polyamide N-ethyl-glycinyl entity (Equation 1) linked to the bases via a $\left[CH_{2}-CO\right]$ amide bond to the glycinyl nitrogen (N^2^).

(1) $$\left[-N^{1}H-CH_{2}-CH_{2}-N^{2}H-CH_{2}-C\left(=O\right)-\right]$$

These novel analogues contain the same number of backbone atoms (6), and atoms linking the backbones to the bases (3), like their natural counterparts (Figure S2, in the Supplementary Materials); the N- and C-terminals corresponding to the 5′- and 3′-ends of DNA respectively. Poly-PNAs were readily synthesized using conventional solid-phase peptide chemistry [15]. Remarkably, the Danish researchers found that complementary PNAs formed stable duplexes, forming Watson–Crick base-paired double helices in a similar manner to DNA and RNA [16]. Moreover, PNA strands also formed stable duplexes, via Watson–Crick base-pairings, with their DNA and RNA counterparts [17,18]. However, PNA sequences containing more than 25 bases were found to be insoluble in water, so despite some remarkably similar attributes to natural nucleic acids, increasing insolubilities shown by all long PNAs (>25-mers) prevents them from becoming uncharged, alternative COSMIC-LOPERS.

### 2.2. The Importance of Phosphates

In a landmark 1987 paper in Science entitled “Why nature chose phosphates?”, the late Frank Westheimer applied critical physical chemistry analysis to attempt to answer the question [19]. In addition to the diesters in DNA and RNA, other phosphate esters and phosphate anhydrides play essential roles in biochemistry: coenzymes; energy storage (adenosine triphosphate, ATP); creatine phosphate, CP; phosphoenolpyruvate and in metabolic products (e.g., glucose-6′-phosphate and fructose 1,6-diphosphate). It is clearly of great importance for all living entities, from bacteria to blue whales, to retain these essential molecules within their cell membranes, which are dominated by relatively hydrophobic substances like fatty acids. All of these essential compounds are esters or diesters of the trivalent phosphoric acid, which has three ionisable O-H bonds. The first pKa of phosphoric acid and that of mono and diesters is approximately 2, meaning that all the phosphate-containing molecules will be ionized at physiological pH (7.4), and will therefore be trapped within the lipophilic cellular membranes.

Nature’s choice of phosphoric acid instead of alternatives like arsenic, citric, glutamic and silicic acids primarily arises from the fact that phosphate esters are much more stable than their alternative counterparts. Arsenic occurs just below phosphorus, in group 5 of the periodic table and forms many analogous compounds with the same valencies as phosphorus. The first pKa of arsenic acid, 2.19, is very close to that of phosphoric acid. However, arsenate esters are much less stable than their phosphorus counterparts; the di-isopropyl ester is completely hydrolysed in less than 2 min in water at room temperature, whereas the half-time of hydrolysis (t_1/2_) of dimethyl phosphate, in 1M NaOH solution at 110 °C, is about 24 h. Even though arsenate diesters possess a negative charge at physiological pH, the larger arsenic atom is more easily accessible to attacking nucleophiles like water, and so arsenate esters are both kinetically and thermodynamically unstable with respect to aqueous hydrolysis. Moreover, phosphorus is geologically much more abundant, at 1.0 ppth (i.e., 1.0 mg/g) in the Earth’s crust (0.1%) compared to arsenic, which occurs at 5 ppm.

Similarly, the diesters and triesters of silicic acid are much less stable than those of phosphoric acid, despite the much greater geological abundance of silicon. Furthermore, silicic acid is a much weaker acid, with a pKa of 9.50, so it would be mostly unionized at physiological pH, rendering the electrophilic silicon atom much more vulnerable to nucleophilic attack by water molecules than its phosphorus counterpart.

**Figure 5:**
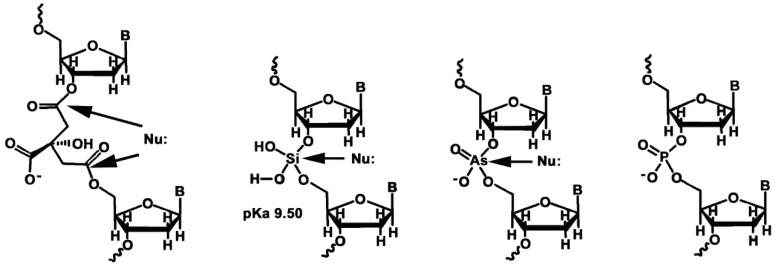
The phosphodiester linkage in nucleic acids is much more resistant to attack by nucleophiles (Nu:) than the alternative arsenate diesters, silicate diesters and citrate diesters.

The only viable trivalent organic acid, citric acid, also forms esters that are much less stable than those of phosphate. The first pKa, of the central carboxylic acid, is 2.92 and so is quite similar to that of phosphoric acid at 2.00 and would thus be ionized at physiological pH. However, a citrate nucleotide would have a longer, 5-atom linker connecting the nucleosides, and the negative charge would provide very little effective shielding of the 5′- and 3′-ester linkers against nucleophilic attack by water. Nature’s choice of anionic phosphate diesters as the backbone of her information storage systems is governed by the fundamental physico-chemical properties of phosphoric acid and its central phosphorus atom. While phosphate diesters and anhydrides are thermodynamically unstable with respect to hydrolysis, the negative charge and the relatively small size of the phosphorus atom (*cf* arsenic) afford them more protection from rapid nucleophilic attack by water, enabling them to persist in an aqueous environment for much longer periods of time than their arsenic, silicon and carboxyl ester counterparts (Figure 5).

### 2.3. Nucleic Acids Containing Acyclic and Non-Ribose Sugar-Phosphate Backbones

#### 2.3.1. Acyclic 3′-1′-glycerol Phosphates

**Figure 6:**
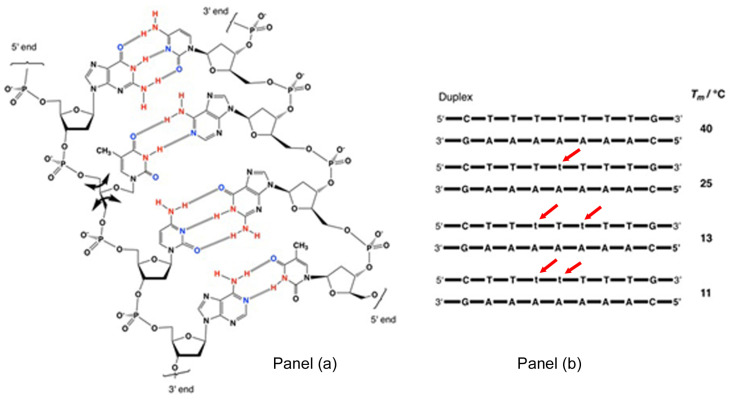
The more flexible 3′-1′-glycerol linker disrupts base-pairing and duplex formation in comparison to the more rigid 2′-deoxyribose panel (**a**), as revealed by the much lower melting temperatures of the duplexes containing one or two of these linkers panel (**b**).

In the late 1980s, Steven Benner’s group (then at the ETH in Zurich) pondered what effect the replacement of ribose sugars with a more flexible open-ended linker would have on the stability of nucleic acid helices. Nucleotides containing the more flexible 3′-1′-glycerol linker were synthesized and incorporated at various positions into synthetic oligodeoxynucleotides nine bases in length, containing the sequence 5′-CTTTTTTTG-3′. The complementary sequences 3′-GAAAAAAAC-5′ were also prepared as they form duplexes held together through Watson–Crick pairing (Figure 6 panel a). The UV-melting temperatures *T_m_* [20] were measured for the standard duplex (which contains only deoxyribose-phosphate) and then for several other duplexes in which one, then two deoxyribose-phosphates were replaced by the more flexible 3′-1′ glycerol units, denoted by t (Figure 6 panel b, red arrows). The results are depicted in Figure 6 and Figure S3 (in the Supplementary Materials). Replacement of one deoxyribose sugar with a glycerol unit lowers the melting temperature by 15 °C. The substituting of two deoxyribose sugars depresses the melting point by 27 °C, and by 29 °C if the two glycerol units are adjacent.

Clearly, this more flexible backbone, with free rotation about the C3′–C2′ and C2′–C1′ bonds, destabilizes duplexes (Figure 6 panel a, thick black arrows). This is because it is thermodynamically unfavourable to constrain it in a relatively rigid structure like a DNA helix, and it indicates that oligonucleotides made from this linker, or Glycerol Nucleic Acids, GNAs, could not be COSMIC-LOPERS.

#### 2.3.2. Hexose Sugar-Phosphates

**Figure 7:**
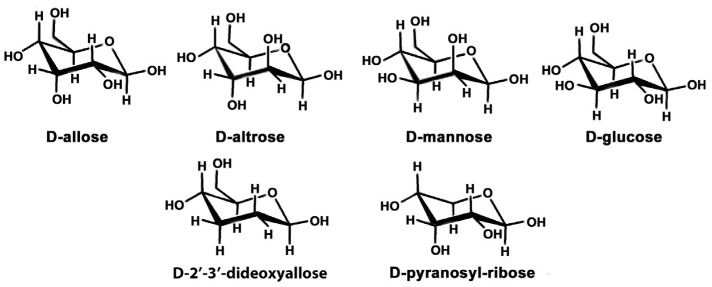
The six hexose sugars used by Eschenmoser’s group to make hexose-nucleic acids.

Beginning in 1986, Albert Eschenmoser’s group, also based at ETH in Zurich, carried out an intensive research programme; synthesizing and studying analogues of DNA and RNA that contain 6-membered ring sugars i.e., hexoses, instead of ribose and deoxyribose [21]. Nucleic acids containing six different hexoses were synthesized, five of which were the naturally occurring D-allose, D-altrose, D-mannose, D-glucose and D-pyranosylribose, and one dideoxy derivative, 2′,3′-dideoxyallose (a hexose equivalent of 2′-deoxyribose) (Figure 7).

They discovered that the hexopyranosyl-(6′-4′) systems, namely (6′-4′)-altro-pyranosyl-, (6′-4′)-allo-pyranosyl-, (6′-4′)-manno-pyranosyl- and (6′-4′)-gluco-pyranosyl showed Watson–Crick base pairing that was greatly inferior in terms of strength and selectivity compared to natural DNA and RNA. In fact, the (6′-4′)-gluco-pyranosyl system does not form duplexes in which complementary sequences exhibit Watson–Crick base-pairing [22]. The reason for this is intra-strand steric hindrances, which occur in the pairing conformations, caused by the 2′- and 3′-OH groups, clashing with the backbone and the edges of the bases respectively. This steric hindrance is especially severe in the (6′-4′)-glucopyranosyl system; graphically displaying the reason why nature’s most abundant carbohydrate, “D-glucose, is not a component of its information storage system”; too much steric hindrance caused by “too many atoms” [23] (Figure S4, in the Supplementary Materials).

In marked contrast, however, the (4′-2′)-ribo-pyranosyl system, (which is the pentapyranosyl isomer of RNA) denoted p-RNA, was shown to be superior to both DNA and RNA, in terms of both duplex stability and the fact that exclusively Watson–Crick base-pairing is observed (Figure S5, in the Supplementary Materials). However, neither Hoogsteen nor reverse Hoogsteen motifs have yet been observed in p-RNA duplexes, and p-RNA sequences will not form stable Watson–Crick duplexes with their DNA or RNA complements. But of all the hexose systems synthesized and studied by Eschenmoser’s group, p-RNA is perhaps the most COSMIC-LOPER like.

Most surprisingly, the 6′-4′ (2′,3′)-dideoxyallopyranosyl system, which is the hexose version of 2’-deoxyribose, (and hitherto referred to as homo-DNA, being a homologue of DNA) displayed distinctly different base-pairing rules to naturally occurring DNA and RNA. Hoogsteen purine-purine pairings, A–A and G–G, were favoured over Watson–Crick A–T pairings, with the base-pairing energies decreasing in the following order: G–C>A–A=G–G>A–T (Figure S6, in the Supplementary Materials). Expanding the 2′-deoxyribose ring by one [-CH_2_-] group to the hexose changes the orientation of the purine bases on the scaffold to such an extent that purine-purine interactions not observed in DNA become manifest and take precedence over Watson–Crick A–T pairings. But, as any good organic chemistry graduate will remind us, 6-membered aliphatic saturated rings are more rigid systems that prefer a “chair conformation” in which all bulky substituents take the “equatorial” positions, in which intra-ring steric repulsions are minimal. An extensive X-ray crystallographic analysis of homo-DNA duplexes (complementary sequences containing 2, 4, 6, 8, 10 and 12 bases), was carried out by Martin Egli’s group in the early 2000s [24]. Their findings revealed that homo-DNA duplexes showed much less helicality and cannot form the more tightly twisted double helices that are observed in DNA and RNA. This appears to be a direct result of the presence of the more rigid hexose sugar, which almost always occurs in the chair conformation. This study also revealed significant differences between the backbone-base inclination angles observed in homo-DNA when compared to both DNA and RNA [25]. In B-form DNA, this angle is 0°; in A-form RNA it is −30° but in homo-DNA, it is +45°. These differences (+45° from DNA, +75° from RNA) prevent homo-DNA from forming complementary Watson–Crick duplexes with either DNA or RNA (*cf*, unlike PNAs; S6). DNA and RNA can form complementary cross-pairs because DNA can adopt the A-form helical geometry.

#### 2.3.3. Alternative Ribose and Hexose-Sugar Phosphates; XNAs

**Figure 8:**
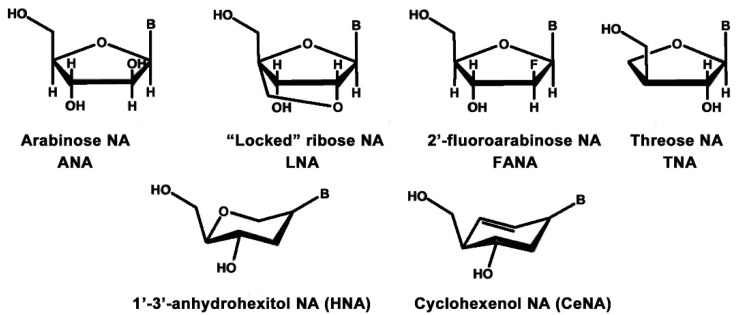
The six xeno-nucleosides, built from 4 pentoses and 2 hexoses used to construct xeno-nucleic acids, XNAs.

In 2012 a significant breakthrough in alternative nucleic acids research was announced. An international team comprising molecular biologists and synthetic organic chemists, led by Phil Holliger and Vitor Pinheiro respectively, based at the Medical Research Council (MRC) Laboratory of Molecular Biology, Cambridge, UK, reported the discovery of synthetic nucleic acids, known as xeno-nucleic acids (XNAs) that were capable of replication and evolution, with the same fidelity as DNA and RNA [26]. The group synthesized and tested XNAs containing six different sugar derivatives; four pentoses and two hexoses. The pentoses were arabinose (the 2′-epimer of ribose), 2′-fluoro-arabinose, threo-furanose and 2′-O, 4′-C-methylene-β-ribose (“locked”-ribose), and the two hexoses were 1, 5-anhydrohexitol and cyclohexenose. Nucleic acids containing arabinose were termed ANAs; 2′-fluoro-arabinose, FANAs; threo-furanose, TNAs; locked-ribose, LNAs; 1,5-anhydrohexitol, HNAs; and cyclohexenose, CeNAs (Figure 8).

All six XNAs were shown to form complementary Watson–Crick paired helices to themselves, and their DNA and RNA complements, including the HNAs (Figure S7, in the Supplementary Materials), previously prepared by Piet Herdewijn as potential anti-sense agents [27]. This is quite remarkable as the 1, 5-anhydrohexitol is an isomer of 2′, 3′-dideoxyallose, used by Eschenmoser et al to construct homo-DNA, which, as has been discussed above, forms distinct duplexes with different pairing rules, and will not cross-pair with DNA or RNA.

**Figure 9:**
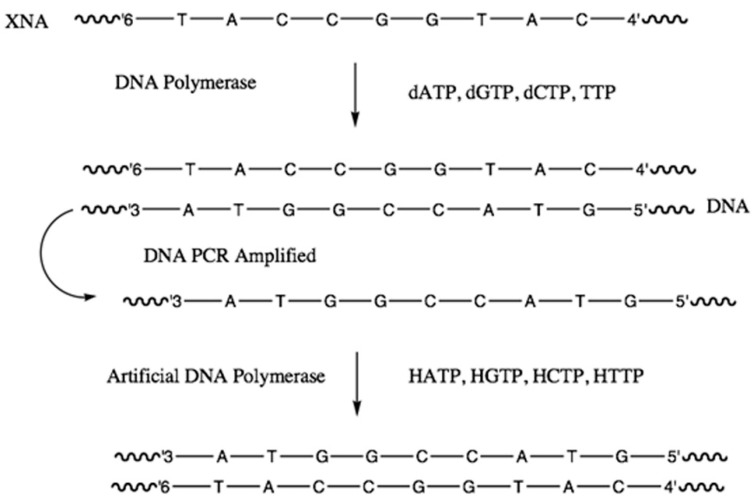
Specially evolved “Artificial” DNA polymerases enable XNA and DNA to act as templates for each other in PCR.

Nucleic acid replication is facilitated by enzymes, known as polymerases. DNA amplification is routinely performed using thermostable enzymes in the polymerase chain reaction (PCR; [28]). This technique has revolutionized molecular biology, and found widespread applications in different fields, from forensics to palaeontology. A true test of an XNA, or indeed any nucleic acid variant’s ability to replicate and evolve, requires that it can undergo PCR-amplification as with its natural counterpart. By employing a technique called in-vitro evolution [29], Holliger et al were able to synthesize specially evolved polymerases that accepted the XNA-triphosphates and incorporated them into new complementary XNAs and amplify XNA duplexes using PCR. Furthermore, some of these polymerases were able to synthesize XNAs from a complementary DNA template, and likewise synthesize DNA from an XNA template (Figure 9). All six XNAs showed high replication fidelities, ranging from 95% in LNAs to as high as 99.6% in HNA and CeNAs (Figure S7, in the Supplementary Materials).

#### 2.3.4. Catalytic XNAs: Xenoribozymes (XNAzymes)

In 2015, Holliger et al [30] reported the syntheses of a series of XNA oligomers, which they called XNAzymes, hereafter referred to as “xenoribozymes” that displayed catalytic behaviours similar to ribozymes. Their xenoribozymes used four of their non-standard sugar backbones, namely the previously mentioned ANA, FANA, HNA and CeNA.

Xenoribozymes were generated using an elaborate in-vitro selection method. Initially, xenoribozymes displaying RNA-cleavage ability (endonuclease) were prepared using all four backbones. The endonuclease activities included both intra-molecular (cis which refers to cleavage of an RNA segment in an RNA–XNA chimaera) and bimolecular (trans-cleavage of a phosphodiester bond in a bound RNA substrate). The FANAzymes displayed reaction rates comparable with analogous ribo- and deoxyribozymes, but the ANA-, CeNA- and HNAzymes all displayed rates that were 2–600 times slower.

### 2.4. Nucleic Acids Containing Non-Standard Nucleobases

The past three decades have seen the generation of synthetic nucleic acids having additional nucleobase “letters” that form additional nucleobase pairs that are distinct from the naturally occurring A–T/U and G–C. These new pairings can be divided into two different groups; conventional non-standard base pairs, developed by Steven Benner, use alternative hydrogen-bonding patterns to those observed in A–T/U and G–C; and unconventional non-standard base pairs, reported by the groups of Eric Kool, Ichiro Hirao and Floyd Romesberg, which are hydrophobic by nature and cannot interact by hydrogen-bonding.

The presence of additional nucleobase pairs expands the sequence and functional diversity of nucleic acids. A six-letter genetic alphabet has 6^n^ different sequences of length n and an eight-letter alphabet 8^n^. These varied expanded genetic systems have led to the development of new molecular biology tools, clinical diagnostic kits, and, very recently, artificial Darwinian genetic systems and the potential for the development of synthetic life.

#### 2.4.1. Nucleic Acids Containing Conventional Non-Standard Bases

Beginning in the late 1980s, the group of Steven Benner began an intensive research programme to develop an expanded genetic information system (AEGIS) that incorporated additional nucleobase letters into nucleic acids. By carefully examining the structures of the standard nucleobases A, C, G and T/U, they realized that subtle re-arrangement of the embedded ring nitrogens and the appended amino and keto oxygen functionalities could generate 8 new nucleobases; arranged as 4 new base-pairs which obeyed the Watson–Crick paradigm; hydrogen bonding between acceptors and donors, and large purines binding with small pyrimidines. As these new bases display different hydrogen-bonding arrangements they can only pair with their complementary partners in the same way that adenine only pairs with thymine or uracil: and guanine only pairs with cytosine. Benner’s group were intrigued as to why nature uses only 4 nucleobases and pondered whether additional nucleobase letters were used in the precursor RNA world and whether alien biochemistries might utilize different nucleobases, and possibly even expanded genomes.

**Figure 10:**
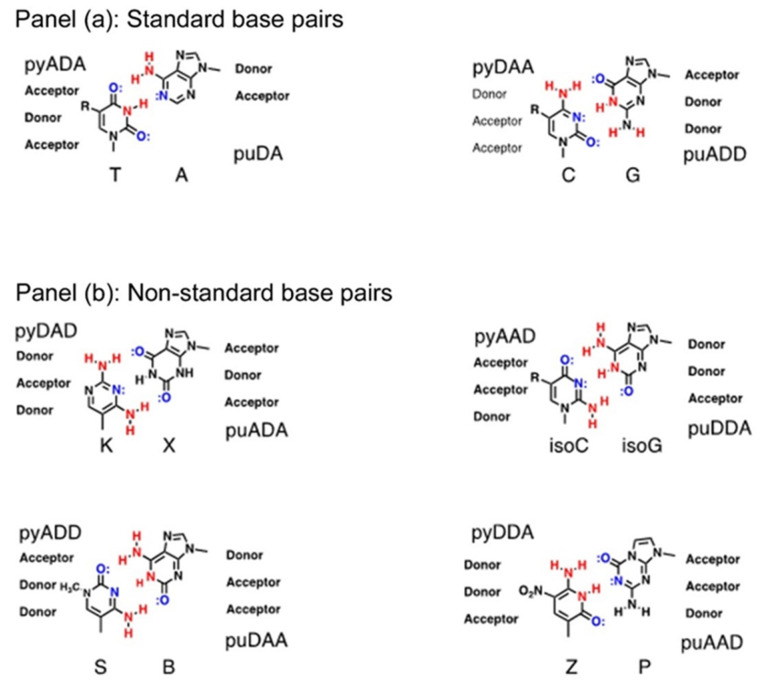
Panel (**a**) shows standard base pairs in contemporary biology. Panel (**b**) shows an Expanded Genetic Information System containing 8 non-standard bases arranged as 4 non-standard pairs; K–X, isoC–isoG, S–B and Z–P.

Iso-cytosine and iso-guanine are isomers of the naturally occurring cytosine and guanine bases respectively, in which the exocyclic amino and keto oxygens are inter-converted. Xanthine is the product of hydrolysis of guanine; it occurs naturally as the biosynthetic precursor to both theobromine and caffeine. Its partner, 2,4-diamino-pyrimidine, is linked to deoxyribose or ribose sugars via a carbon atom, and is a C-nucleoside, and has been given the trivial name *kappa* (K). The two other non-standard pyrimidines: 3-methyl-6-amino-pyrimidin-2-one, referred to as S, and 6-amino-5-nitro-1H-pyridin-2-one, known as Z, also form C-nucleosides (Figure 10).

Synthetic DNA and RNA duplexes containing one or more non-standard base pairs were prepared and extensively studied and shown to be at least as stable as their natural counterparts. Indeed, in a 12-mer duplex (Figure S8, in the Supplementary Materials), when a G–C pair is replaced with an isoG–isoC, the melting temperature remains the same at 52 °C. When G–C is replaced by K-X, the melting temperature increases to 58 °C, and to 62 °C when replaced by the Z–P pair. Because the non-standard bases will not cross-pair with any of the standard bases, oligonucleotides containing them tend not to form duplexes with standard oligonucleotides that possess a similar standard base sequence. Cross-pairing of oligonucleotides that contain similar but mismatched sequences is a common problem with diagnostic kits that utilize nucleic acid hybridization assays and can generate many false-positive signals, thus greatly reducing their detection efficacies.

Clever use of oligonucleotides containing non-standard base pairs can greatly reduce these problems, and greatly improve the efficacies and detection limits of nucleic acid diagnostics [31]. In 1995 Bayer launched a branched DNA (bDNA) Quantiplex diagnostics probe, whose schematic setup is shown in (Figure S9, in the Supplementary Materials). The chip contains a series of oligonucleotide capture probes that are complementary to 15–20 bases on the target nucleic acid, e.g., mRNA from HIV or HBV. A set of chimeric oligonucleotides containing both standard and isoC/isoG rich sequences then bind to their complements on both the captured analyte (via the standard base sequence), and with non-standard bDNA sequences via the isoC/isoG containing sequences, anchoring them to the chip. Finally, another set of isoC/isoG oligonucleotides containing fluorescein-labelled isoG conjugates are then captured through complementary binding to sequences on the bDNA components. This incorporation of non-standard (isoC/isoG) bases into the parts of the hybridization complex that does not interact with the analyte greatly improves the detection efficacy as it prevents contaminant nucleic acids (from the sample) from binding and disrupting the signalling; thus, greatly improving the affinity of the system for the target analyte. This probe can detect as few as 50 viral mRNA molecules per ml of blood from HIV and HCV patients and is widely used to monitor the progress of these patients on both existing and new antiviral drug treatments [32,33]. More recent versions of this diagnostic system have been used to detect viral RNA from Zika and coronaviruses [34].

DNA polymerases, both wild-type and artificial, have been widely used to construct oligonucleotides containing all of the non-standard base pairs, placing them opposite their partners in the template strands with >95% efficiency. Following on from this, PCR systems capable of replicating AEGIS nucleic acids, including those with several repeating sequences of non-standard bases, were also developed. These systems worked best with the Z–P and S–B pairs. However, the isoC–isoG pair was shown to possess two significant disadvantages. Firstly, isoG has a minor tautomer that enables it to pair with thymine, and so after successive rounds of PCR, the isoC–isoG base pair is progressively edited out, replaced by the standard A–T pair (Figure S10, in the Supplementary Materials). Secondly, the iso-cytidine nucleoside is highly acid-sensitive, rendering the syntheses of isoC rich oligonucleotides difficult. Such inherent issues with the isoC–isoG pair, like those observed for the 6-membered homo-DNA, display clearly why nature’s nucleic acids don’t contain them (Figure S11, in the Supplementary Materials) [35].

In early 2019, the Benner group, in collaboration with four other leading research groups, announced the discovery of “hachimoji” DNA and RNA [36]; a genetic system with eight letters (hachimoji being the Japanese word for eight). The 8-letter nucleic acids contained the Z–P and S–B pairs in addition to the standard pairs. Complementary 16-mer duplexes containing these two pairs in many varied sequences were crystallized and analyzed and found to adopt the DNA B-form, with similar major and minor groove widths. Benner remarked that these “hachimoji DNAs met the Schrodinger requirement for a living system, forming the same ‘aperiodic crystal’ regardless of the sequences”.

Hachimoji DNA sequences were then transcribed into hachimoji RNAs using a T7 RNA polymerase variant (Y639F H784A P266L, “FAL”). A hachimoji variant of the spinach fluorescent RNA aptamer was then designed, with one Z–P and one S–B pair incorporated, and transcribed from hachimoji DNA using the T7 RNA polymerase FAL variant. The standard aptamer folds and binds the ligand 3,5-difluoro-4-hydroxybenzylidene imidazolinone (DFHBI), which fluoresces green. The hachimoji variant was shown, by circular dichroism (CU) experiments to maintain the natural aptamer’s folded structure. Further analyses confirmed the presence of the non-standard base pairs in the correct positions. In addition, the substitution of U at position 50 with Z yielded, as expected, an aptamer variant that did not fluoresce in the presence of the ligand, DFHBI, as the Z is close enough to the bound ligand to enable quenching of its fluorescence. This is therefore the first Darwinian genetic system built from eight rather than the standard four letters, and its’ potential applications are limitless.

In 2018, Benner and co-workers reported the discovery of two new DNA-like systems that supported hydrogen-bond molecular recognition on a backbone that violated the size complementarity, small pyrimidine pairs with large purine, paradigm [37]. These two new nucleic acid systems are referred to as “skinny” where the base pairs are small 6-membered ring pyrimidines and “fat” where they are large 9-membered ring purines. Both skinny and fat pairs obey Watson–Crick hydrogen-bond complementarity (Figure S12, in the Supplementary Materials). Three pyrimidine pairs are possible, namely S–Z, T–K/K’ and C–V—where S and Z are the non-standard pyrimidines that were previously used in conventional non-standard base pairings with their purine partners B and P respectively. Naturally occurring thymine pairs with both kappa (denoted here by K’) and 3-nitro-2,6-diaminopyridine (K). Two ‘fat’ pairs are possible as in D–X and B–P—where D is diaminopurine, the 2′-amino derivative of adenine; and X is 7-deazaxanthosine and was preferred over xanthosine, as it lacks the 7-nitrogen, preventing D–X Hoogsteen interactions from occurring. B is isoguanosine as its N-1 tautomer.

Melting temperature studies of both fat and skinny 15-mer duplexes clearly revealed that they do form the expected Watson–Crick hydrogen-bonded systems. The skinny duplexes had sharp ‘melting’ temperatures (*T*_m_) that averaged 58.3 °C, higher than those of standard Watson–Crick analogues (~36 °C), even when the A residues in the latter were replaced by D (~45 °C) which forms 3 hydrogen-bonds to T. The fat duplexes also displayed sharp melting but at higher temperatures (~77.7 °C). Both skinny and fat DNA systems disfavoured mismatches, which were seen to lower the melting temperatures by 3–5 °C, which is similar to what is observed in standard DNA duplexes (Figure S13, in the Supplementary Materials).

X-ray crystallography studies were performed on 16-mer duplexes containing four central skinny (5′-CTTATA**KKTT**TATAAG) and six central fat (5′-CTTAT**XXXDDD**ATAAG) complementary base pairs. The obtained data confirmed that the skinny and fat regions do indeed interact via Watson–Crick hydrogen bonding, and although the duplex geometry in these regions was neither A- nor B-form, the standard portions of the duplexes exhibited the B-form.

#### 2.4.2. Unconventional Non-Standard Bases

“Unconventional” non-standard nucleobases refer to hydrophobic aromatic analogues of the standard 4 bases that lack hydrogen bond acceptor and donor functional groups, and so are unable to interact via Watson–Crick hydrogen-bonded base pairings. The “nonpolar nucleoside isosteres” concept, was introduced by Eric Kool in 1994 [38] with the introduction of 2,4-difluorotoluene, F, which is a near-perfect isostere (shape mimic) for naturally occurring thymine (Figure S14, in the Supplementary Materials). Kool’s group also used other toluene-nucleoside derivatives as thymine replacements and indoles and benzimidazoles as purine substitutes. The inability of difluorotoluene to partake in Watson–Crick hydrogen bonding with adenine was revealed, unsurprisingly, by lower melting temperature values for complementary duplexes which contained A–F in place of A–T or G–C (Figure S15, in the Supplementary Materials). Despite possessing three lone pairs of electrons, fluorine hardly ever acts as a hydrogen bond acceptor in organic molecules [39]. The highly electronegative F atom holds these lone pairs very tightly, and in fact, organic molecules rich in fluorine, fluorocarbons, are even more hydrophobic than their hydrocarbon counterparts. Very surprisingly, Kool et al. then revealed that difluorotoluene could act as a replacement for thymine during the syntheses of complementary DNA duplexes by several A- and Y-family type DNA polymerases [40]. The DNA pol I, Klenow fragment (Kf) from *E. coli* inserted dATP opposite F and dFTP opposite A in DNA primer-templates with similar efficiency as the natural A–T base pair. Full-length products were obtained from templates containing up to eight F residues, although this did not work when two or more Fs were in sequence. Similar results were obtained with other toluene derivatives such as 2, 4-dichloro-, 2-fluoro 4-chloro- and 2-bromo 4-chloro-toluene. These results attracted much scrutiny from the biochemical community, and much criticism [41], to which Eric Kool responded [42] most eloquently. Prior to this work, the widely held view about enzyme-catalysed DNA replication was that hydrogen bonding was the dominant factor that governed how the enzyme placed the correct base in the synthesis strand opposite its partner in the template. Kool’s work strongly suggests that this is not so and that steric effects, governed by base shape, is the paramount source of DNA replication fidelity, and indeed, since 2002, editions of the classic biochemistry text of Stryer have cited these findings [43]. In 1998, Kool and his coworkers reported the first hydrophobic base-pair (Figure S16, in the Supplementary Materials), difluorotoluene-4-methylbenzimidazole (F–Z’) [44]. 4-Methylbenzimidazole is referred to here as Z’ to avoid confusion with the Benner group base 6-amino-5-nitro-1H-pyridin-2-one, which is also denoted as Z. 4-Methylbenzimidazole is a non-polar isostere of adenine lacking the 3-nitrogen. This study revealed that DNA pol I Klenow fragment (Kf) efficiently inserted F and Z’ opposite each other, as well as F–A and Z’–T and was the first to show that a purely hydrophobic base-pair could be replicated in this way. This inspired a flurry of activity in unconventional hydrophobic non-standard base-pair research, led by the groups of Ichiro Hirao and Floyd Romesberg.

In 2006, Hirao’s group reported the development of an expanded genetic system using a hydrophobic base pair between 7-(2-thienyl)-imidazo[4,5-b]pyridine (Ds) and pyrrole-2-carbaldehyde (Pa). This pair maintained the Watson–Crick shape complementarity, with Ds acting as an isostere of adenine, and Pa an isostere of thymine. Initial problematic misincorporations, of dDsTP opposite template Ds, and dATP opposite template Pa were overcome by using the respective γ-amidotriphosphates. This led to the first-ever PCR amplification of an expanded genetic system containing a hydrophobic base pair, using vent DNA polymerase (exo) which proceeded with very high efficiency [45]. The same paper reported the successful transcription of DsTP and PaTP into RNA, with very high selectivity and efficiency, using T7 RNA polymerase. However, the presence of two or more of these unnatural bases in sequence in primer-template results in greatly diminished yields of full-length product, and PCR will not replicate such primers. Subsequently, another pyrrole derivative, 2-nitro-4-propynylpyrrole, known as Px, was shown to be as efficient in PCR systems as Pa but without the cross-pairing problems inherent to the latter, and replaced it as the smaller hydrophobic base [46]. The Hirao group’s principal application of their hydrophobic base pair has been on the development of novel DNA aptamers with enhanced binding efficacies for proteins (Figure S17, in the Supplementary Materials), arising from hydrophobic interactions between Ds and Px with hydrophobic amino acid side chains [47].

Beginning in 1998 at the Scripps Institute in La Jolla, the group of Floyd Romesberg began an extensive program aimed at the development of an artificial genetic system containing hydrophobic non-standard bases [48]. Many different hydrophobic base pairs were synthesized, incorporated into DNA templates, and the synthetic efficacies of many naturally occurring, and artificial DNA polymerases were assessed. Two base pairs, dNaM-d5SICS and dNaM-dTPT3, proved to be superior and were incorporated into synthetic strands opposite their template partners with the same efficacies as the naturally occurring base pairs (Figure S18, in the Supplementary Materials). However, the presence of two or more in sequence reduced the synthetic efficiency.

X-ray crystallographic studies on complementary duplexes containing these pairs clearly revealed that they interact via hydrophobic stacking, and this distorts the helicity of the duplex when two or more are in sequence, and this has profound effects on the ability of polymerases to produce full-length complementary products.

PCR systems were developed that successfully amplified primers containing these non-standard base pairs, with the same efficiency as the natural pairs following 100 or more cycles [49]. However, they did not work when two or more of these non-standard bases are in sequence in the template. In a breakthrough in 2013, the d5SICS-dNaM pair were then inserted into a bacterial plasmid which was taken up by a strain of *E. coli* which was shown to replicate only in the presence of the unnatural nucleoside triphosphates d5SICS-TP and dNaM-TP in addition to the natural ones; dATP, dCTP, TTP and dGTP. This was the first-ever report of an organism that could grow and replicate with an unnatural base pair in its genome [50].

The Romesberg group continued work on the development of semi-synthetic organisms (SSO) that can use a 6-letter genetic alphabet to make proteins containing unnatural amino acids [51,52]. Using the dTTP3-dNaM pair, they cleverly engineered an *E. coli* containing the unnatural base pair in its’ genome, mRNA and tRNA. Use of tRNA-loading enzymes that were tolerant of unnatural anticodons was vital to the success of this project. Beginning with the *E. coli* tRNA synthetase that loads serine onto tRNA, they inserted serine into a position on the green fluorescent protein (GFP) via a codon containing one of the non-standard bases paired with its partner on the anticodon. They then engineered a modified *E. coli* whose gene for tRNA synthetase was replaced with a gene for an enzyme from a different microbe, *Methanosarcina barkeri* which uses the rare amino acid pyrrolysine, and the expressed tRNA synthetase added pyrrolysine into the same position on GFP. They then went a step further and inserted a gene from the archaea *Methanococcus jannaschi* for a tRNA synthetase which adds the even rarer amino acid 4-azido phenylalanine, and the expressed enzyme added 4-azido phenylalanine at the same position in GFP, encoded by the non-standard base containing codon.

This work paves the way for the development of both semi and fully synthetic microbes, containing an expanded 6-letter genetic alphabet, that can be engineered to produce novel proteins containing non-standard amino acids, both rare and synthetic, with great potential for the development of new materials and therapeutics.

## 3. Conclusions

This article has summarized the discoveries and developments in de novo nucleic acid research going back just over 30 years. The results have greatly increased our understandings of naturally occurring nucleic acids DNA and RNA and have expanded the scope of molecular structures that can support life, both here on Earth in new, artificial Darwinian systems, and potentially elsewhere in the Universe. In this respect we can indeed state that life may not necessarily be limited to the nucleic acid building blocks seen in terran DNA and RNA; alternatives to ribose and deoxyribose, like arabinose, threo-furanose, locked-ribose, 1,5-anhydrohexitol and cyclohexenose, are feasible. Non-standard nucleobase pairs like Z–P and S–B which display Watson–Crick size and hydrogen bonding complementarities, could replace the naturally occurring A–T and G–C pairs or indeed occur together with them in alien organisms with expanded genetic alphabets. In addition, non-standard nucleobase pairs which follow Watson–Crick hydrogen bonding but not size complementarity are possible; three “skinny” pyrimidine-pyrimidine pairings; S–Z, T–K/K’ and C–V; and two “fat” purine-purine pairs; D–X and B–P. Furthermore, unconventional hydrophobic base pairs like Ds-Px, 5SICS-NaM and TPT3-NaM might be possible, as long as they are separated by standard base-pairs and not adjacent in a sequence. The possibility of exclusively hydrophobic base pairs occurring in an alien genetic system, which has arisen in a non-aqueous solvent, is also conceivable.

We may safely conclude then that extraterrestrial life if it has ever arisen elsewhere in the Universe, might not necessarily use DNA or RNA, but, in an aqueous environment like Earth, it will have a sugar-phosphate backbone in its genetic information storage biopolymers.

## Supplementary Materials

The following are available online at https://www.mdpi.com/2075-1729/10/12/346/s1. Figure S1. (a) The numbering system used for nucleosides, in which the sugars use primed numbers 1’-5’ in order to distinguish them from the bases. (b) A section of an oligonucleotide strand; RNA when X=OH and R=H; DNA when X=H and R=CH_3_. The sequences shown here are GUCA (RNA) or GTCA (DNA). Figure S2: the structure of peptide nucleic acid (PNA) compared to DNA. Figure S3: 3’-1’-glycerol nucleic acids (GNA) are flexible linker analogs of DNA. Figure S4: nucleic acids containing D-allose, D-altrose, D-mannose and D-glucose cannot form Watson–Crick base-paired duplexes because of severe intra-strand steric clashes, arising from interactions between the 2’-OHs and the edges of the bases (right arrows) and the 3’-OHs and the backbone (left arrows). Figure S5: 4’-2’-Pyranosyl RNA forms very stable duplexes with exclusively Watson–Crick base-pairings. Figure S6: D-2’-3-Dideoxyallose nucleic acid (homo-DNA) duplexes display distinct Hoogsteen type base pairing rules very different to those occurring in DNA and RNA, with A-A and G-G base pairs that are more stable than A-T; pairing rules in order of stability G–C > A–A~G–G > A–T; 6’-(A)_3_(G)_3_-4’ duplexes have a melting temperature of 34 °C. Figure S7: XNAs like HNA form complementary Watson–Crick base paired duplexes; with themselves and complementary DNA and RNA sequences. Figure S8: comparison of melting temperatures (*T_m_*) of 12-mer duplexes containing a non-standard base pair X-Y. Figure S9: oligonucleotides containing the non-standard base pair isoC-isoG are used in a Bayer bDNA diagnostic system and greatly improve the detection limits for viral mRNA and other disease-related nucleic acid analytes. Figure S10: isoG has a minor tautomer that pairs with T, resulting in a replacement of the isoG-isoC pair with T-A following PCR amplification. Figure S11: PCR amplification of a 6-letter template containing two adjacent non-standard bases (P-P) [35]. Figure S12: pyrimidine-pyrimidine pairs obey Watson–Crick hydrogen-bonding complementarity, via S-Z, T-K and C-V pairs to generate ‘skinny’ DNA-like duplexes; purine-purine pairs do likewise, via D-X and B-P pairs to generate ‘fat’ DNA-like duplexes. Figure S13: melting temperatures of complementary ‘fat’ and ‘skinny’ and standard DNA duplexes. Figure S14: difluorotoluene is a very good shape mimic for thymine, even though it is much less polar and cannot engage in hydrogen bonding. Figure S15: replacement of thymine (T) with difluorotoluene (F) in an otherwise complementary 12-mer duplex lowers the melting temperature (*T_m_*) significantly. Figure S16: The first reported hydrophobic base pair, difluorotoluene (F)-4-methylbenzimidazole (Z’). Figure S17: The hydrophobic base pairs Ds-Pa and Ds-Px, developed by the Hirao group. Figure S18: The hydrophobic base pairs d5SICS-dNaM and dTPT3-dNaM, developed by the Romesberg group.
