# Supplementary material for: De Novo Nucleic Acids: A Review of Synthetic Alternatives to DNA and RNA That Could Act as Bio-Information Storage Molecules
Source: Life (Basel). 2020 Dec 11;10(12):346. doi: 10.3390/life10120346 (PMC7764398; doi:10.3390/life10120346)
Supplement: Supplementary file 1 [file life-10-00346-s001.pdf]

life

Supplementary Materials

De Novo Nucleic Acids: A Review of Synthetic Alternatives to DNA and RNA That Could Act as Bio-Information Storage Molecules †

Kevin G Devine [1] and Sohan Jheeta [2,*]

1 School of Human Sciences, London Metropolitan University, 166-220 Holloway Rd, London N7 8BD, UK; K.Devine@londonmet.ac.uk
1 Network of Researchers on the Chemical Evolution of Life (NoR CEL), Leeds BD11, UK
* Correspondence: sohan@sohanjheeta.com
† This paper is dedicated to Professor Colin B Reese, Daniell Professor of Chemistry, Kings College London, on the occasion of his 90th Birthday

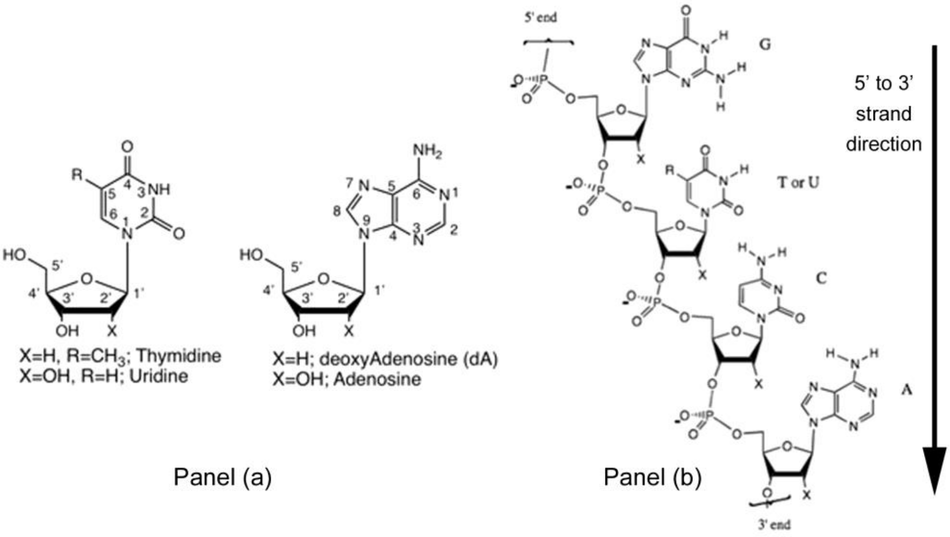

Figure S1. (**a**) The numbering system used for nucleosides, in which the sugars use primed numbers 1′-5′ in order to distinguish them from the bases. (**b**) A section of an oligonucleotide strand; RNA when X=OH and R=H; DNA when X=H and R=CH_3. The sequences shown here are GUCA (RNA) or GTCA (DNA).

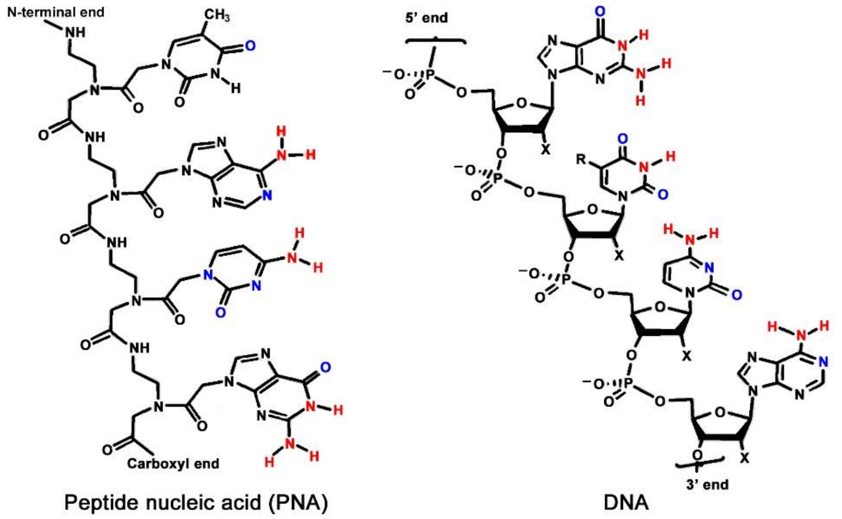

Figure S2. the structure of peptide nucleic acid (PNA) compared to DNA.

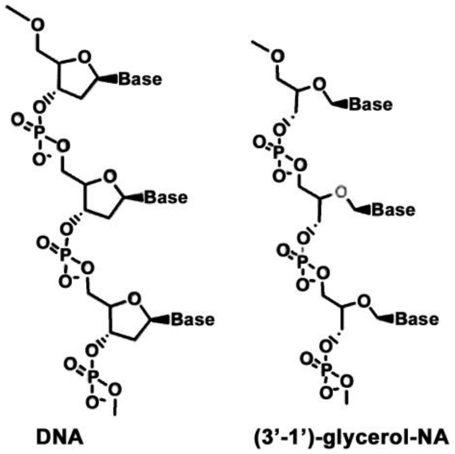

Figure S3. 3′-1′-glycerol nucleic acids (GNA) are flexible linker analogs of DNA.

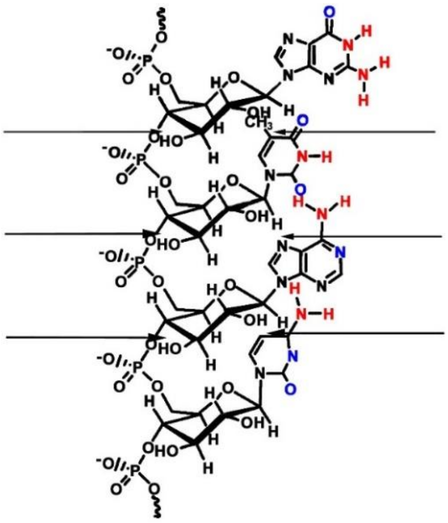

Figure S4. nucleic acids containing D-allose, D-altrose, D-mannose and D-glucose cannot form Watson-Crick base-paired duplexes because of severe intra-strand steric clashes, arising from interactions between the 2′-OHs and the edges of the bases (right arrows) and the 3′-OHs and the backbone (left arrows).

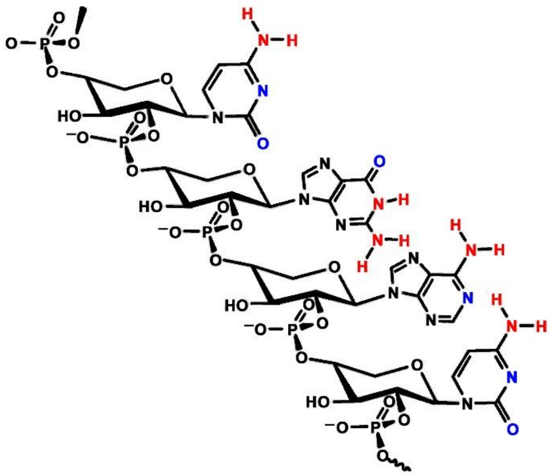

Figure S5. 4′-2′-Pyranosyl RNA forms very stable duplexes with exclusively Watson-Crick base-pairings.

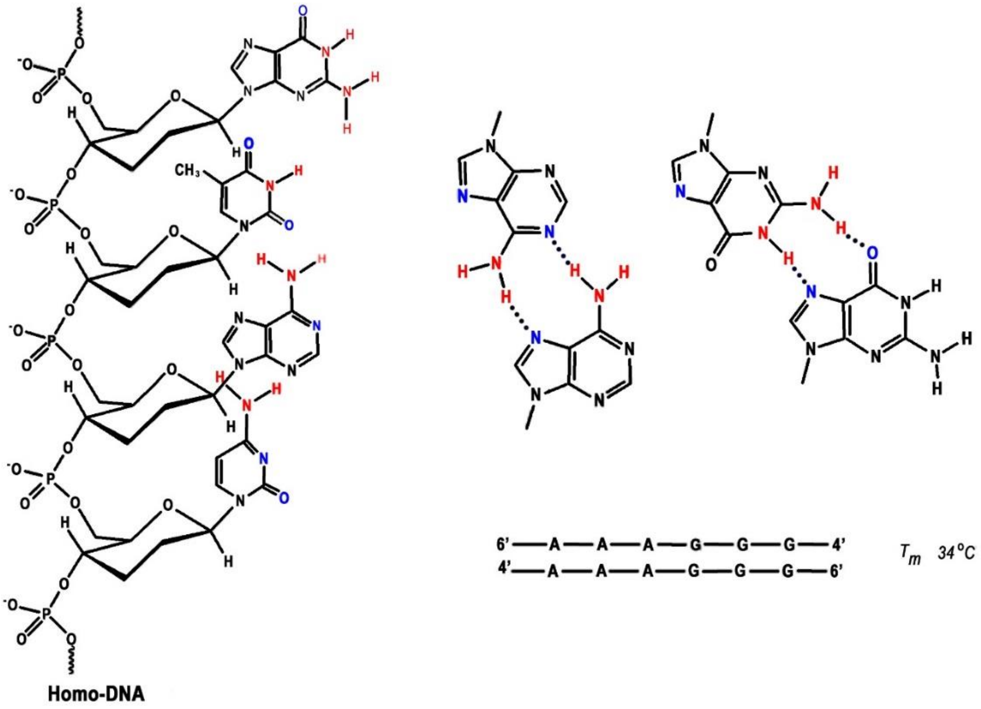

Figure S6. D-2′-3-Dideoxyallose nucleic acid (homo-DNA) duplexes display distinct Hoogsteen type base pairing rules very different to those occurring in DNA and RNA, with A-A and G-G base pairs that are more stable than A-T; pairing rules in order of stability G-C > A-A~G-G > A-T; 6′-(A)3(G)3-4′ duplexes have a melting temperature of 34°C.

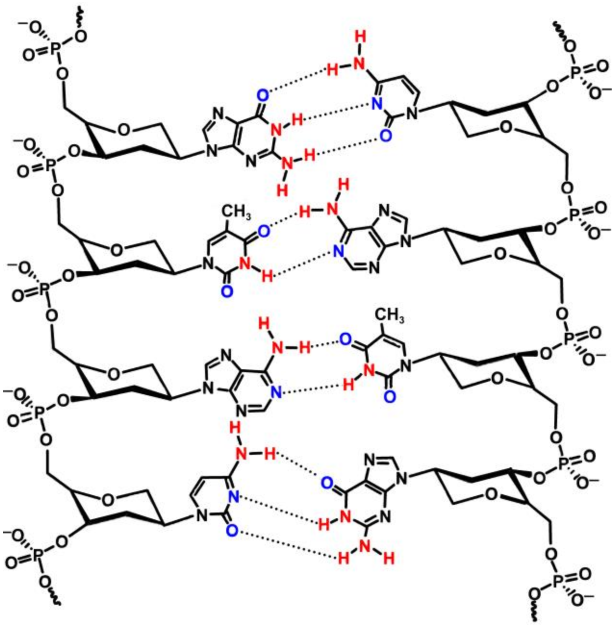

Figure S7. XNAs like HNA form complementary Watson-Crick base paired duplexes; with themselves and complementary DNA and RNA sequences.

5'—C—T—T—T—C—X—C—T—C—C—C—T—3'

3'—G—A—A—A—G—Y—G—A—G—G—G—A—5'

X	Y	T_m /°C
G	C	52
isoG	isoC	52
K	X	58
Z	P	62

Figure S8. comparison of melting temperatures (T_m) of 12-mer duplexes containing a non-standard base pair X-Y.

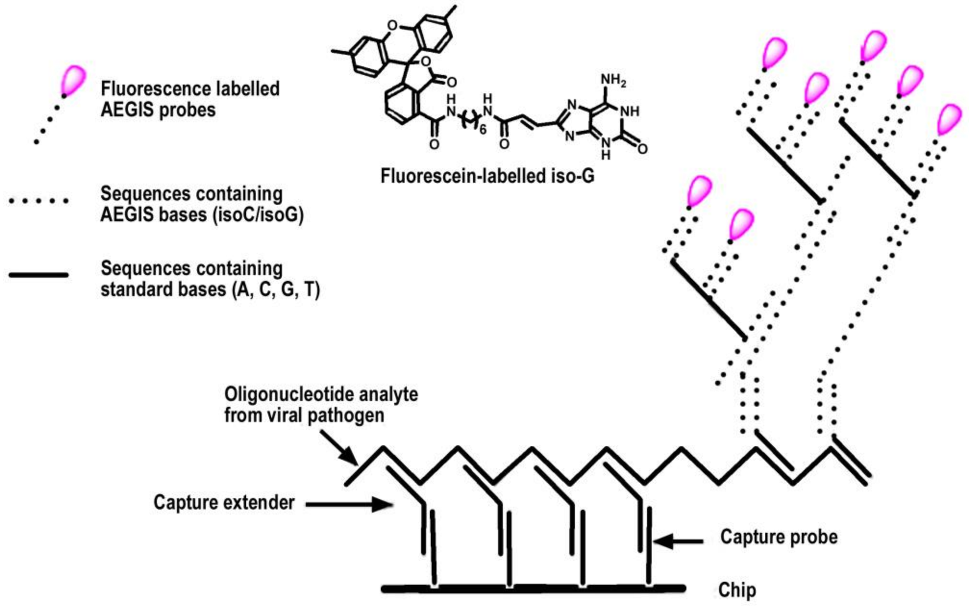

Figure S9. oligonucleotides containing the non-standard base pair isoC-isoG are used in a Bayer bDNA diagnostic system and greatly improve the detection limits for viral mRNA and other disease-related nucleic acid analytes.

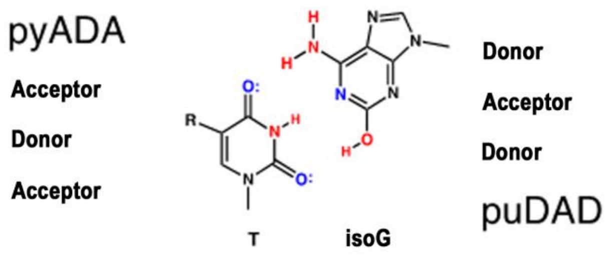

Figure S10. isoG has a minor tautomer that pairs with T, resulting in a replacement of the isoG-isoC pair with T-A following PCR amplification.

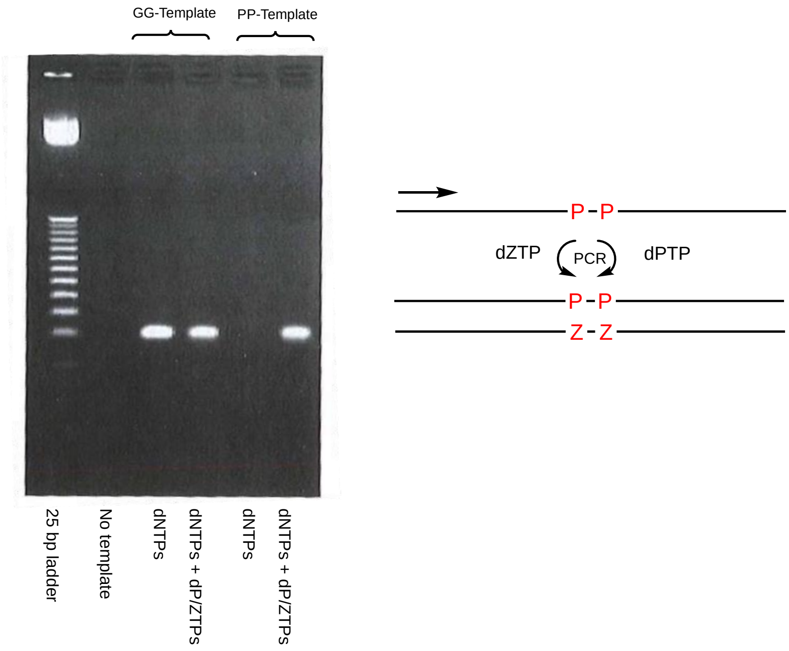

Figure S11. PCR amplification of a 6-letter template containing two adjacent non-standard bases (P-P) (Benner 2011).

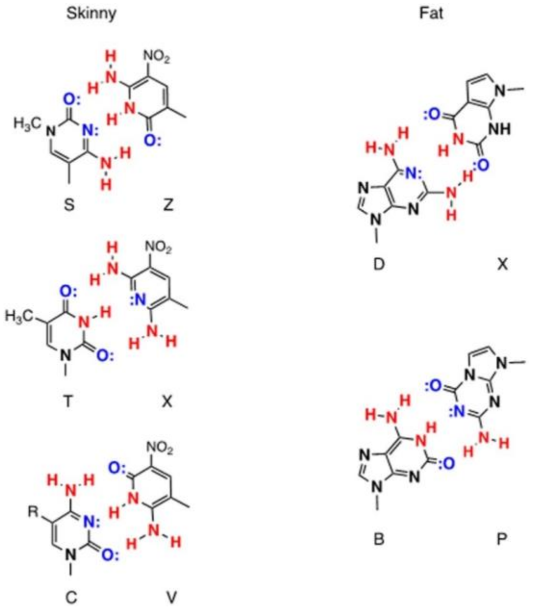

Figure S12. pyrimidine-pyrimidine pairs obey Watson-Crick hydrogen-bonding complementarity, via S-Z, T-K and C-V pairs to generate 'skinny' DNA-like duplexes; purine-purine pairs do likewise, via D-X and B-P pairs to generate 'fat' DNA-like duplexes.

description	sequence pair	T_m (°C)
standard all Watson–Crick geometry	5′-AGA GAA AAA GGA GGA<br>3′-TCT CTT TTT CCT CCT	36.5 ± 1.1
standard all Watson–Crick geometry	5′-AGG AGG AAA AAG AGA<br>3′-TCC TCC TTT TTC TCT	34.8 ± 0.6
3-H-bond all Watson–Crick geometry	5′-DGD GDD DDD GGD GGD<br>3′-TCT CTT TTT CCT CCT	46.3 ± 1.0
3-H-bond all Watson–Crick geometry	5′-DGG DGG DDD DDG DGD<br>3′-TCC TCC TTT TTC TCT	44.9 ± 1.0
AEGIS all fat geometry	5′-**DBB XBP DXX DDP XPX**<br>3′-**XPP DPB XDD XXB DBD**	77.7 ± 0.5
AEGIS all skinny geometry	5′-**KZZ TZS KTT KKS TST**<br>3′-**TSS KSZ TKK TTZ KZK**	58.3 ± 0.9
mispairs, all fat geometry	5′-DBD BXX DDX BPD PPX<br>3′-XBB PXP DBB PXP XXD	<20
mispairs, all fat geometry	5′-DBB XBP DXX DDP XPX<br>3′-XBB PXP DBB PXP XXD	<20
mispairs, all skinny geometry	5′-KZZ TZS KTT KKS TST<br>3′-TZZ STS KZZ STS TTK	<20
mispairs, all skinny geometry	5′-KZK ZTT KKT ZSK SST<br>3′-TZZ STS KZZ STS TTK	<20

Figure S13. melting temperatures of complementary 'fat' and 'skinny' and standard DNA duplexes.

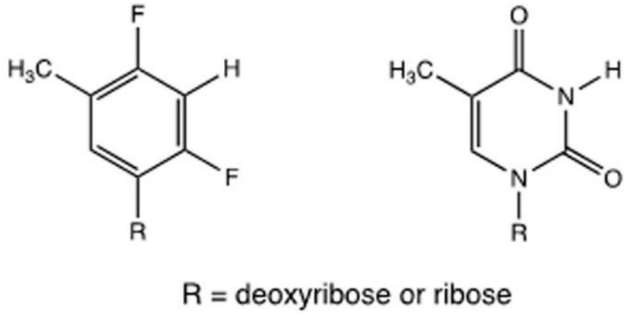

Figure S14. difluorotoluene is a very good shape mimic for thymine, even though it is much less polar and cannot engage in hydrogen bonding.

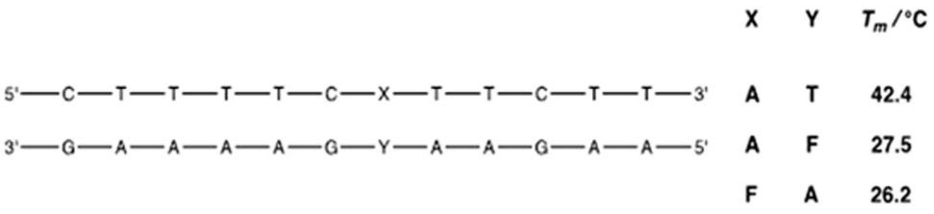

Figure S15. replacement of thymine (T) with difluorotoluene (F) in an otherwise complementary 12-mer duplex lowers the melting temperature (T_m) significantly.

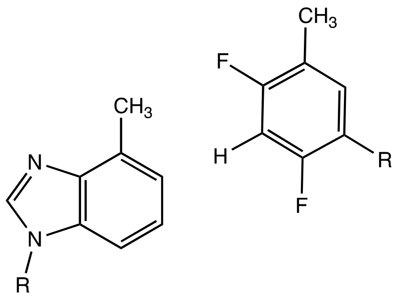

Figure S16. The first reported hydrophobic base pair, difluorotoluene (F)-4-methylbenzimidazole (Z′).

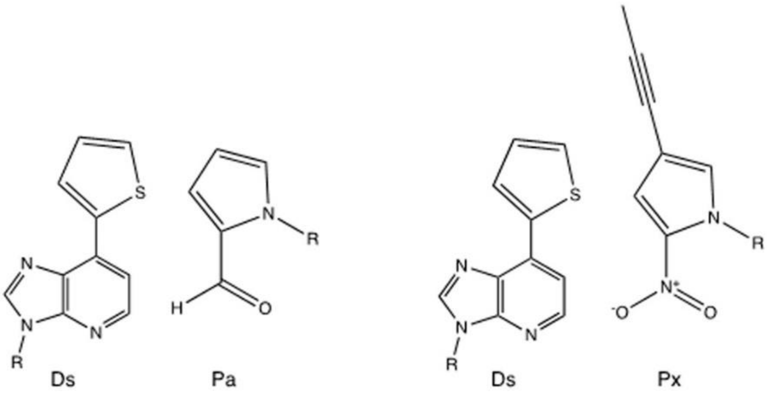

Figure S17. The hydrophobic base pairs Ds-Pa and Ds-Px, developed by the Hirao group.

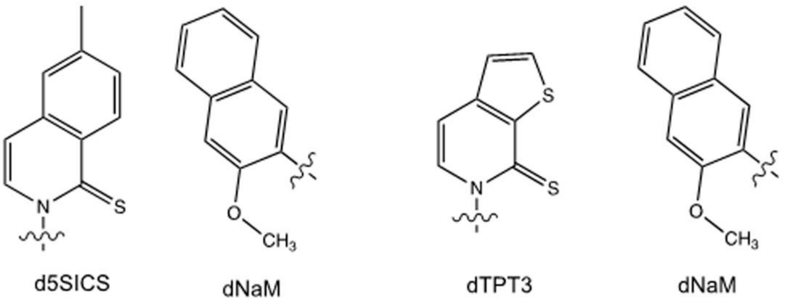

Figure S18. The hydrophobic base pairs d5SICS-dNaM and dTPT3-dNaM, developed by the Romesberg group**.**

Publisher's Note: MDPI stays neutral with regard to jurisdictional claims in published maps and institutional affiliations.

© 2020 by the authors. Licensee MDPI, Basel, Switzerland. This article is an open access article distributed under the terms and conditions of the Creative Commons Attribution (CC BY) license (http://creativecommons.org/licenses/by/4.0/).
